# Inflammatory Myofibroblastic Tumor of the Left Sphenoid and Cavernous Sinus Successfully Treated with Partial Resection and High Dose Radiotherapy: Case Report and Review of the Literature.

**DOI:** 10.7759/cureus.328

**Published:** 2015-09-21

**Authors:** Brandon C Gabel, Mary Goolsby, Lawrence Hansen, Hoi Sang U

**Affiliations:** 1 Department of Neurosurgery, University of California, San Diego; 2 Department of Pathology, University of California, San Diego

**Keywords:** myofibroblastic tumor, plasma cell granuloma, fractionated radiotherapy, steroids, mri, cavernous sinus, tolosa-hunt syndrome

## Abstract

Inflammatory myofibroblastic tumors, also known as plasma cell granulomas or inflammatory pseudotumors, are uncommon lesions that are known to arise in many areas of the body. They are uncommonly found in the skull base region where effective treatment can be difficult. Steroids and radiation therapy with gross total excision when possible remain the treatments of choice. However, the dosing of radiation remains controversial and many patients develop relapse despite medical management. We present the case of a patient who had an inflammatory myofibroblastic tumor of the sphenoid bone and cavernous sinus. He underwent partial surgical resection and transient steroid therapy. This was followed by high-dose fractionated radiotherapy. The patient demonstrated significant resolution in symptomatology and evidence of disease-free progression on repeat imaging.

## Introduction

Inflammatory myofibroblastic tumors, also known as plasma cell granulomas, or inflammatory pseudotumors, are uncommon lesions that are known to arise in many areas of the body. They are uncommonly found in the skull base region where effective treatment can be difficult [1-8].

In general, inflammatory myofibroblastic tumors represent a unique diagnostic challenge. On the one hand, they represent a neoplastic process, whereas on the other hand, they are related to an inflammatory-like syndrome [9-11]. These lesions typically have a large mixture of inflammatory cell infiltrates, including T-cells, B-cells, and macrophages on a bed of myofibroblastic tissue that has a benign tumor-like quality on histopathological analysis.

Inflammatory myofibroblastic tumors involving the skull base, and specifically, the cavernous sinus region, can present with numerous signs and symptoms [7]. They include headache, exophthalmos, thrombosis of the cavernous sinus, carotid artery compression, and cranial neuropathies.

Treatment of these tumors outside the skull base has typically been gross total excision, when possible, and supplemental steroid therapy [6, 12]. Most patients do well with complete resection. However, this is not always possible, especially in cases abutting vital structures such as cranial nerves, cavernous sinuses, and carotid arteries.

Several studies have shown disease-free progression with steroid use [7]. However, steroids are not without side effects, especially in patients with co-morbid medical conditions. In addition, the effects of steroid therapy on pseudotumor involving the orbit and skull base are not always robust or predictable, and frequently high dose steroids are needed to control disease flairs [6, 12-13].  

Radiation therapy as a means of reducing the dependence on steroids and in preventing disease recurrence has been employed. The Darrouzet group presented a patient with inflammatory myofibroblastic tumor of the skull base who underwent biopsy, three months of steroid treatment, followed by radiation using 20 Gy in 10 fractions over 12 days [1]. They have followed this patient for two years, and he has remained in remission. Another case report by Foubert-Samier, et al. has illustrated disease-free progression of Tolosa-Hunt syndrome for nearly eight years out with low dose radiotherapy [14].

However, not all studies using radiation therapy have proved as effective. Lee, et al. treated six patients with both steroid and low-dose radiation therapy and found that tumor recurred in five of the patients [6]. Although low-dose radiation therapy has been proven effective in patients with pseudotumor of the orbit, recurrence rates can be high [15]. Given the vital structures that these lesions often involve, care has been taken to avoid high-dose radiation in the aforementioned studies.

We present the case of a patient who had an inflammatory myofibroblastic tumor of the sphenoid bone and cavernous sinus. He underwent partial surgical resection and transient steroid therapy. This was followed by high-dose fractionated radiotherapy. The patient demonstrated significant resolution in symptomatology and evidence of disease-free progression on repeat imaging. 

## Case presentation

Informed patient consent was obtained prior to treatment for this patient.

**Figure 1:**
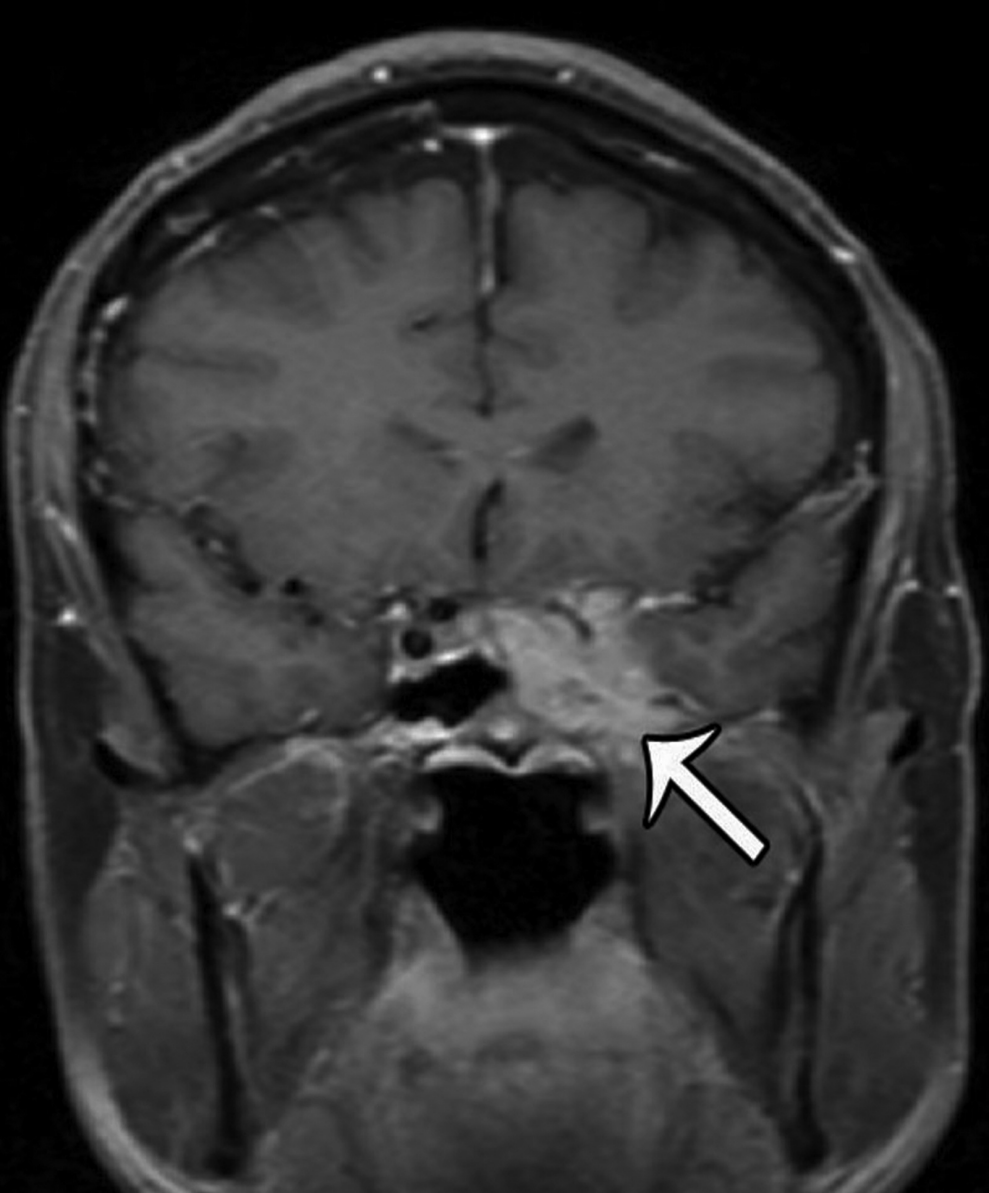
Pre-Treatment MRI Coronal T1-weighted, contrast-enhanced image illustrating an enhancing mass involving the left cavernous and sphenoid sinus.

**Figure 2:**
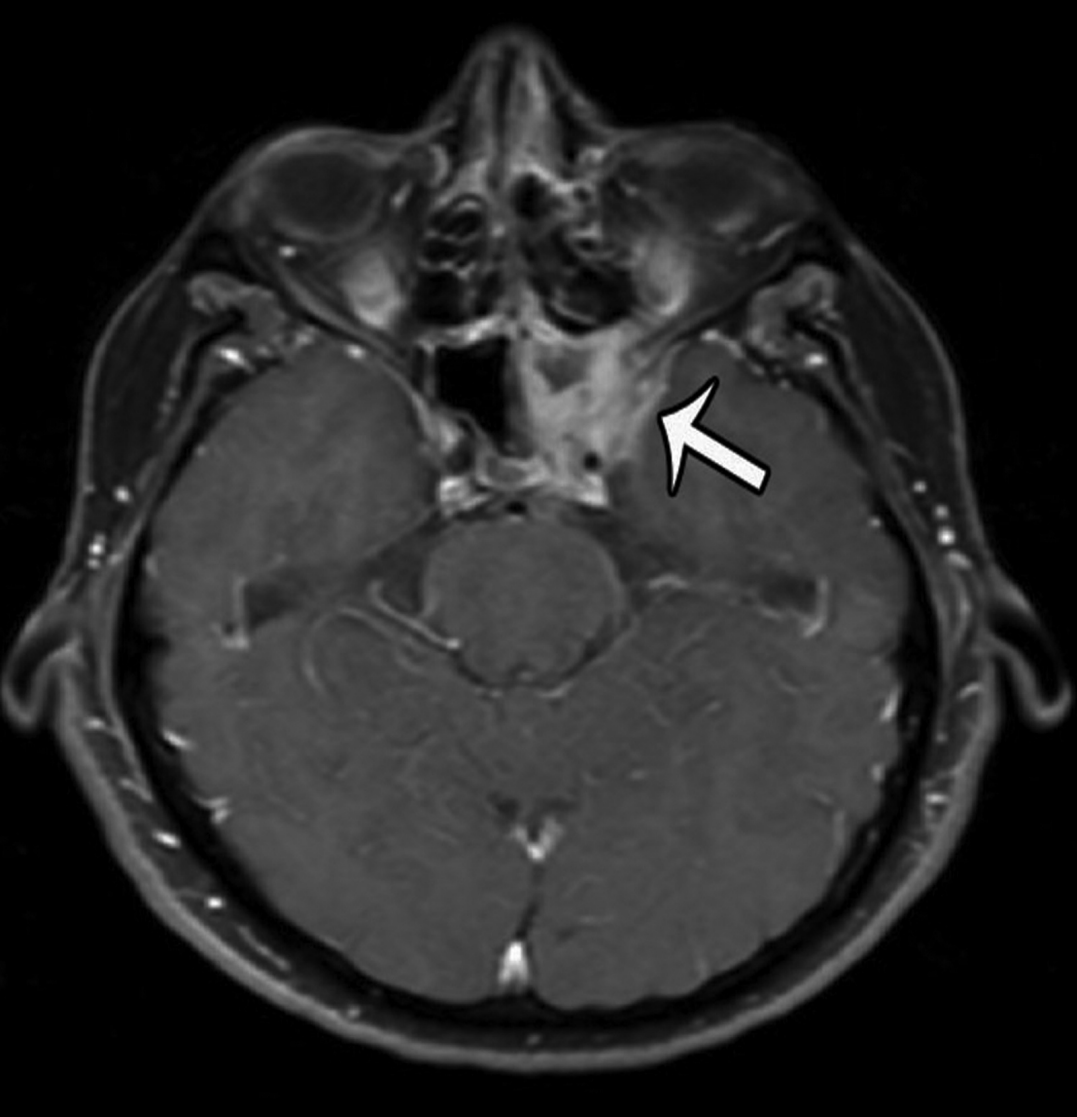
Pre-Treatment MRI Axial T1-weighted, contrast-enhanced image showing the same lesion as in Figure 1.

The patient is a 56-year-old male who initially presented in December of 2011 with a several month history of headaches, paresthesias of the left maxillary region, horizontal binocular diplopia, pressure behind his left eye, and “foggy” vision. MRI imaging revealed a contrast-enhancing lesion involving the left sphenoid and cavernous sinuses (Figures 1-2).

Formal vision testing revealed a left relevant afferent pupillary defect, decreased color discrimination in the left eye, 20/30 uncorrected vision in left eye, as well as an inferior field cut.

The patient underwent a transsphenoidal debulking in January of 2012. Subsequently, the patient’s headaches improved, but his visual disturbances remained unchanged. He was started on prednisone, 20 mg daily, which was tapered off over several months. He also underwent radiation therapy to the residual lesion. His total radiation dose was 45 Gy divided over 25 fractions over a period of five weeks.  

After completion of his radiation and steroid therapy, his vision improved to 20/20 uncorrected and his afferent pupillary defect resolved. In addition, his color vision discrimination testing improved significantly but has not fully returned to normal. He continued to have a mild esophoria with left lateral gaze, but this remains asymptomatic.

The patient was also found to have mild asymptomatic hypogonadism preoperatively. This has remained stable without evidence of other pituitary dysfunction. He has required no hormone replacement therapy.  

**Figure 3:**
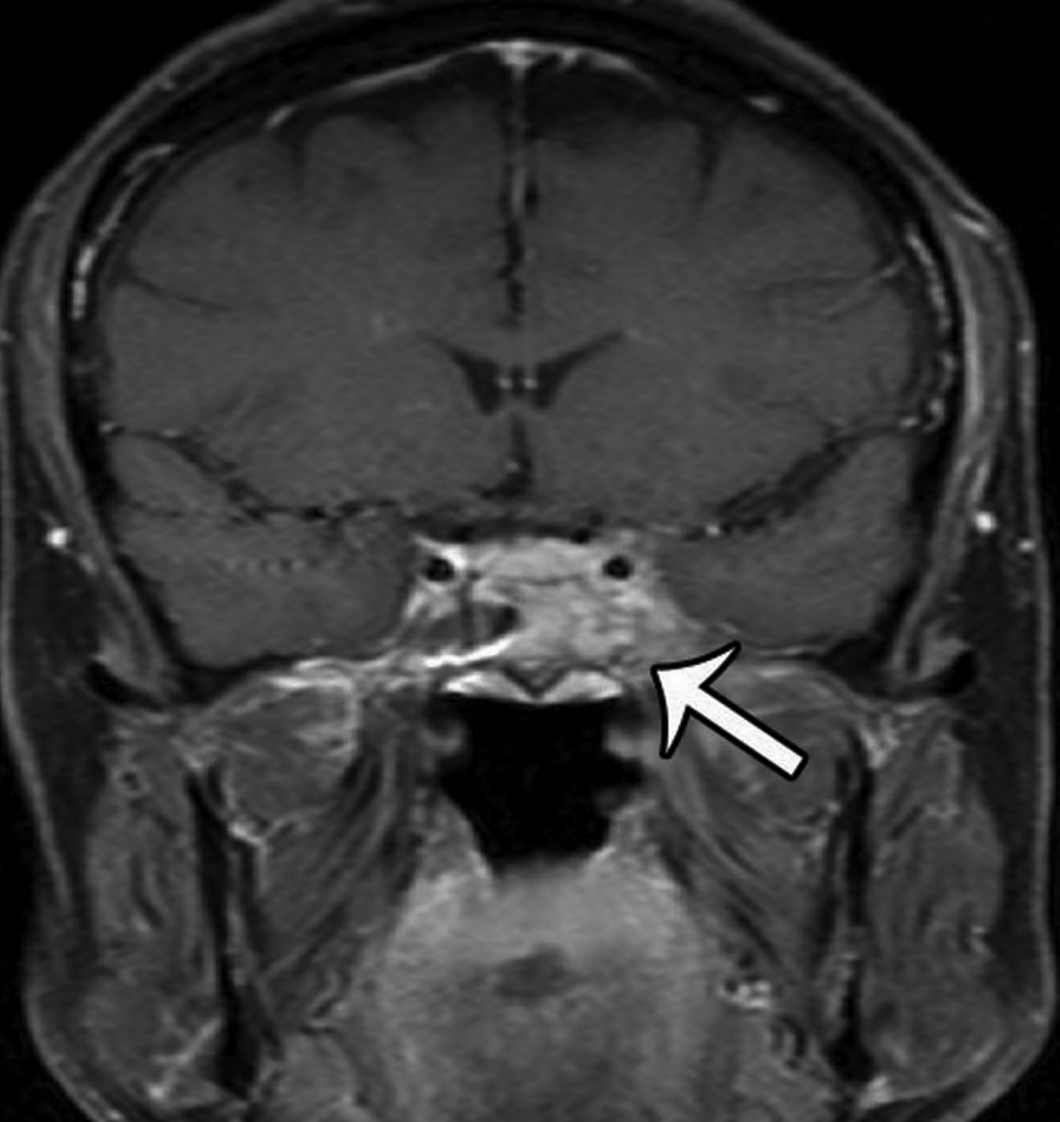
Post-Treatment MRI Coronal T1 weighted, contrast enhanced image illustrating an enhancing mass involving the left cavernous and sphenoid sinus.

**Figure 4:**
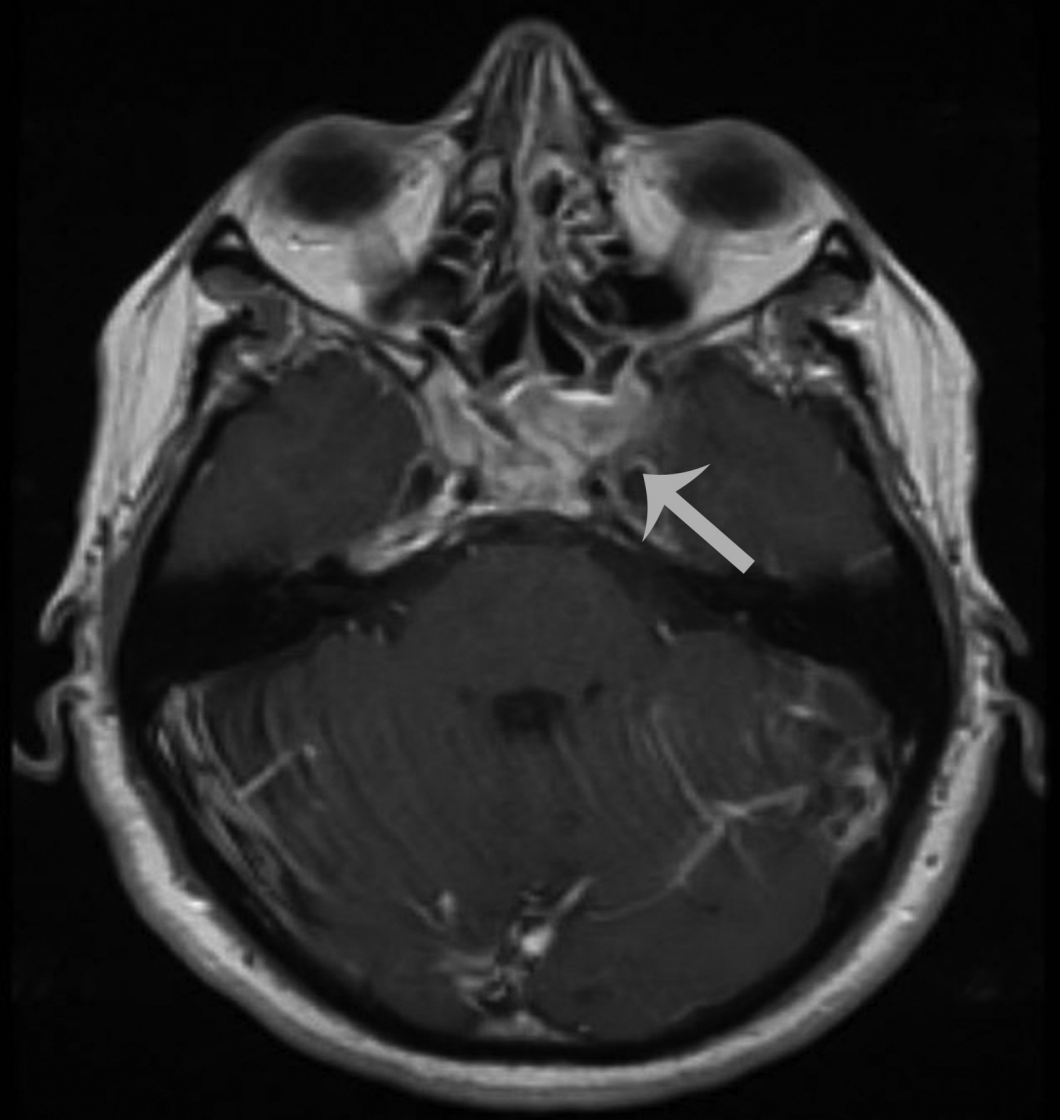
Post-Treatment MRI Axial T1-weighted, contrast-enhanced image showing the same lesion as Figure 3.

An MRI performed 10 months after his initial treatment regimen revealed no new enhancement to suggest progression of his disease (Figures 3-4).

**Figure 5:**
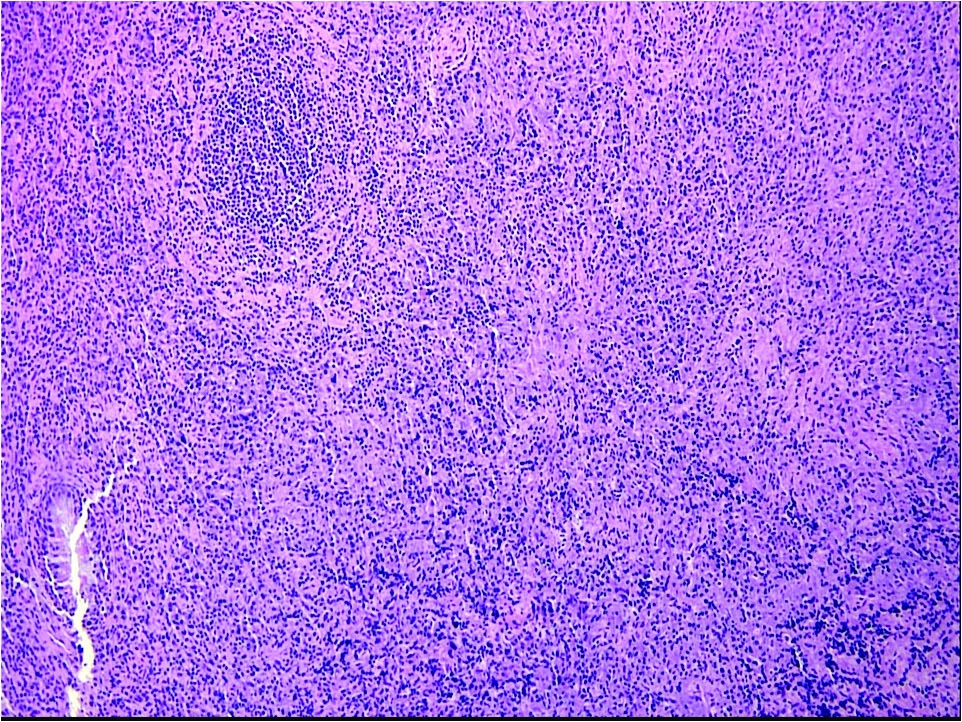
Mixed Inflammatory Infiltrate (Low Power) Dense submucosal mixed inflammatory infiltrate arising in a collagenous background containing haphazard, storiform, or slightly fascicular distributions of fusiform and stellate cells.  Benign mature appearing plasma cells predominate in the chronic inflammatory infiltrate, along with some larger plasmacytoid cells, macrophages, and lymphoid nodules, some of which contain reactive germinal centers. (H&E, 200x).

**Figure 6:**
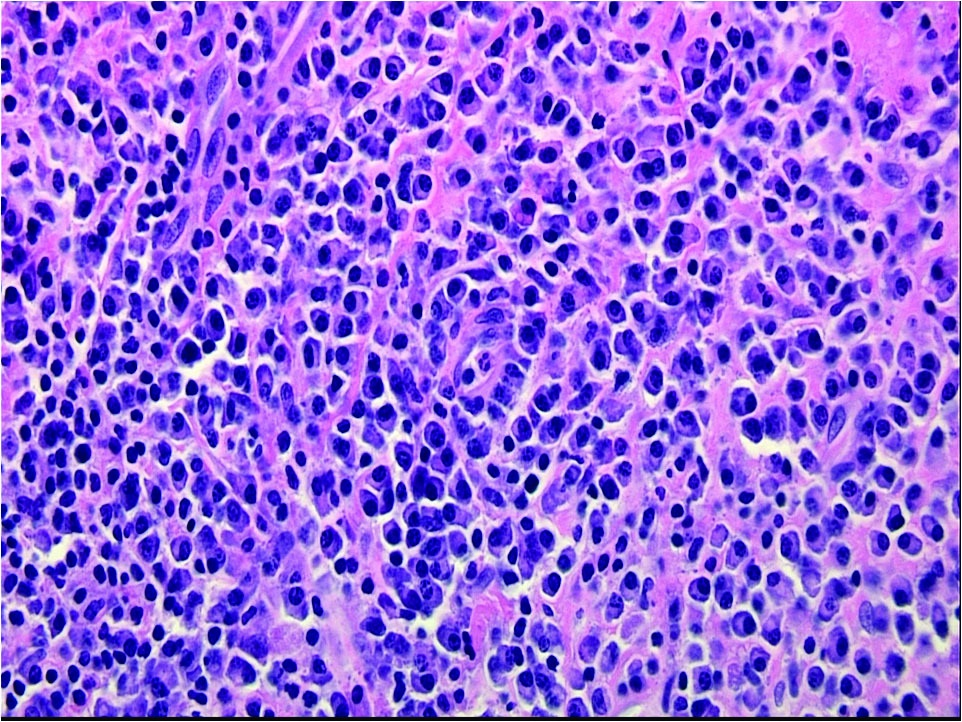
Mixed Inflammatory Infiltrate (High Power) Dense submucosal mixed inflammatory infiltrate arising in a collagenous background containing haphazard, storiform, or slightly fascicular distributions of fusiform and stellate cells. Benign mature appearing plasma cells predominate in the chronic inflammatory infiltrate, along with some larger plasmacytoid cells, macrophages, and lymphoid nodules, some of which contain reactive germinal centers. (H&E, 400x).

**Figure 7:**
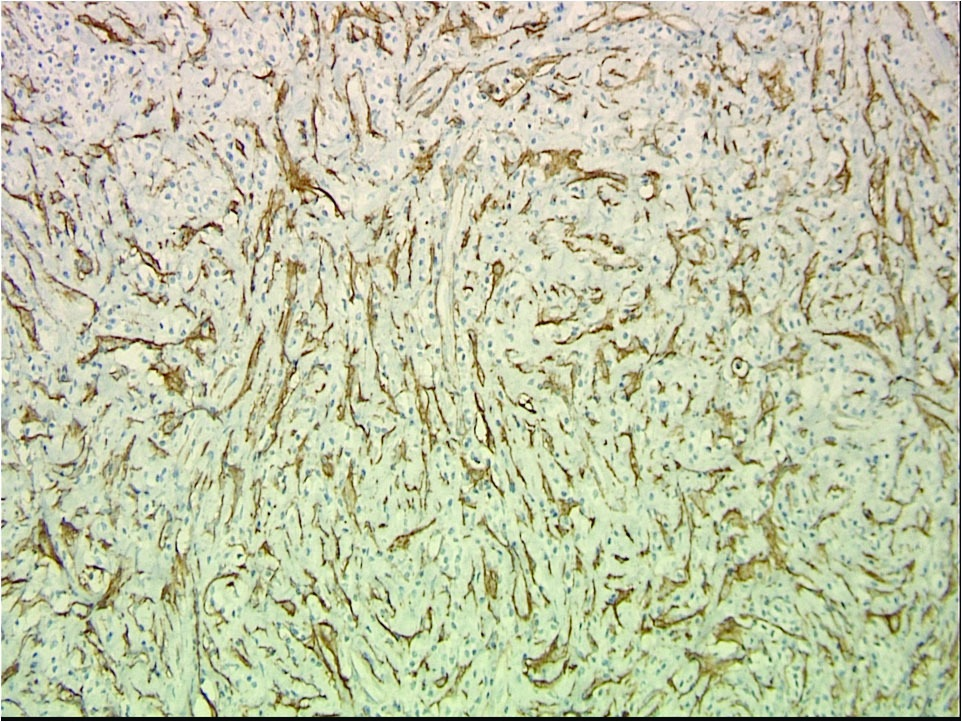
SMA Stain (Low Power) Endothelial cell immunolabeling with smooth muscle actin (SMA) overshadows SMA-positive fusiform spindle cells and some stellate cells which are interpreted as tumor cells. (SMA, 200x).

**Figure 8:**
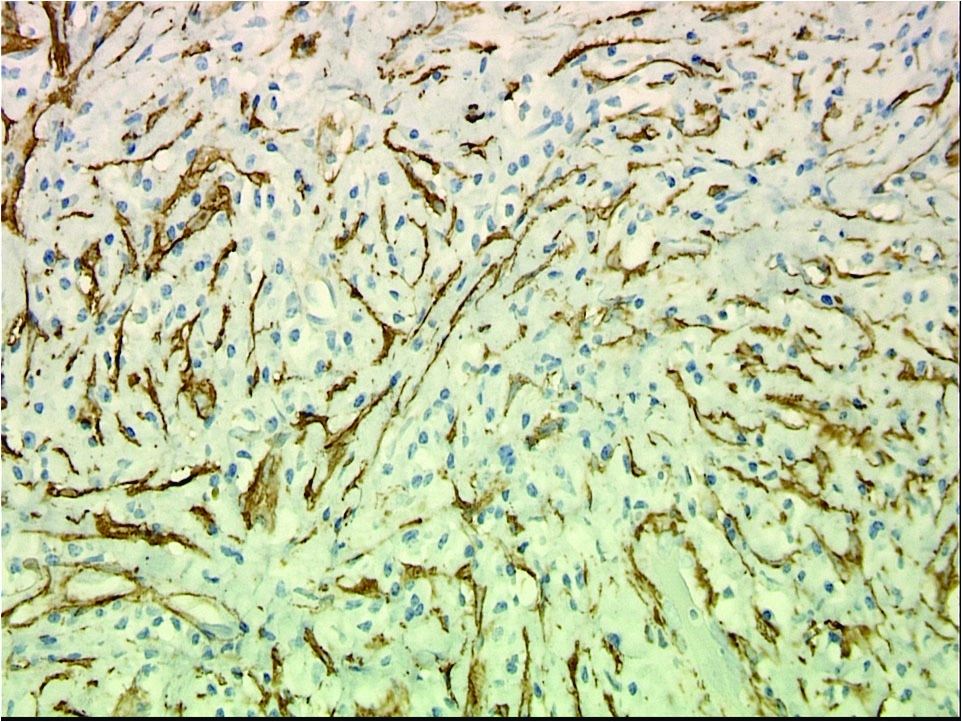
SMA Stain (High Power) Endothelial cell immunolabeling with smooth muscle actin (SMA) overshadows SMA-positive fusiform spindle cells and some stellate cells which are interpreted as tumor cells. (SMA, 400x).

**Figure 9:**
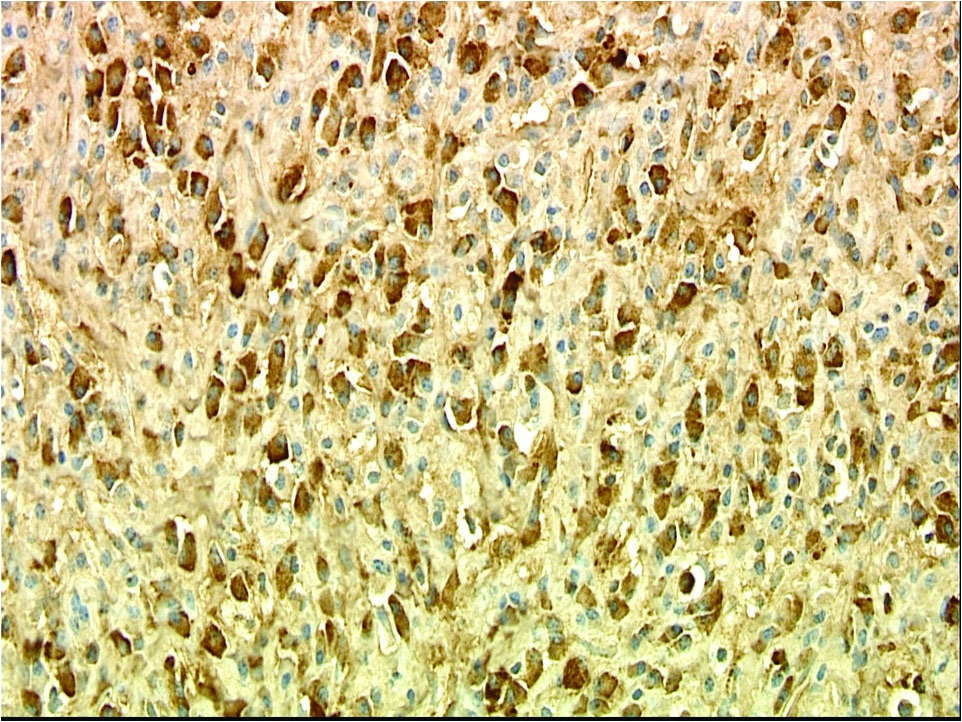
Kappa Light Chain Stain Immunohistochemical stains for kappa and lambda are both strongly positive in numerous cells, indicating a polyclonal reactive proliferation of plasma cells. (Kappa, 400x).

**Figure 10:**
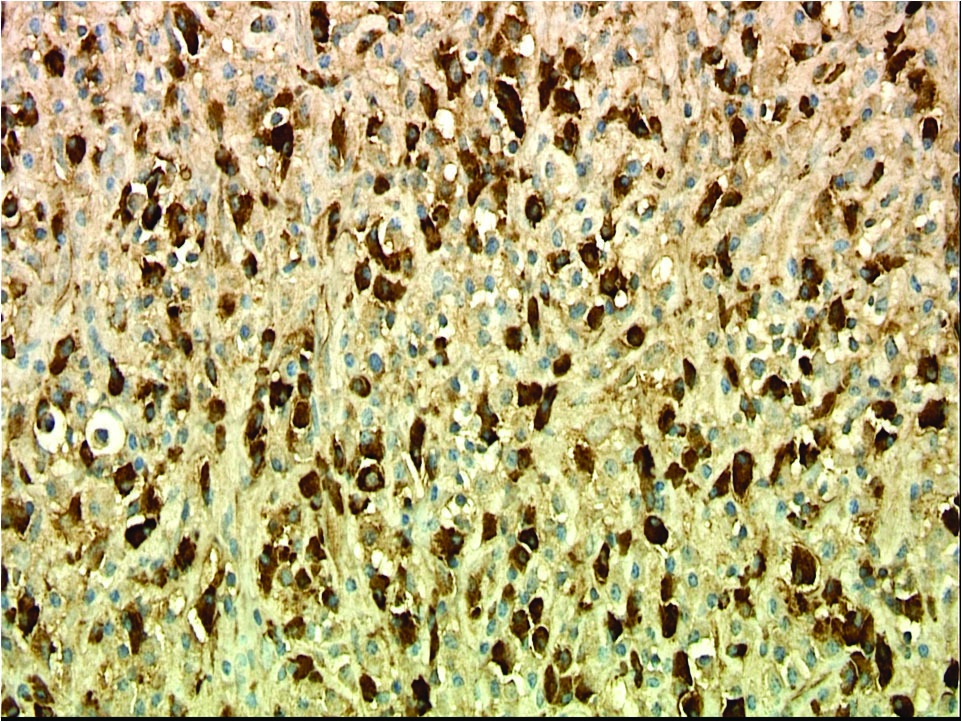
Lambda Light Chain Stain Immunohistochemical stains for kappa and lambda are both strongly positive in numerous cells, indicating a polyclonal reactive proliferation of plasma cells. (Lambda, 400x).

Numerous IgG-positive plasma cells and occasional scattered IgG4-positive plasma cells were identified in the tumor, yielding an IgG4: IgG ratio of less than 10%, which is less than the ratios (from 30% to > 90%) reported for published cases of IgG4 orbital sclerosing disease (Figures 5-6). In addition, there were SMA positive fusiform cells (Figures 7-8) and a small number of histiocytes positive for CD68. A pathological analysis of his tumor stained positive for kappa and lambda indicating a polyclonal source (Figures 9-10). 

## Discussion

We present the case of a patient with an inflammatory myofibroblastic tumor of the left sphenoid and cavernous sinus whose symptoms have improved significantly, and his disease has remained in remission with high-dose radiation therapy.

Most inflammatory pseudotumors of the skull base and orbit are treated with steroid therapy [6, 12]. Although many patients will initially have symptom resolution, not all patients are able to taper off steroids, and some patients continue to have progression of disease despite therapeutic steroid dosing.

This has led many practitioners to recommend radiation therapy as an alternative treatment modality [6, 14-16]. The level of disease control with low-dose radiation therapy remains controversial. Lee, et al. showed significant recurrence despite treatment with steroids and low-dose radiation [6]. However, several case reports have shown good disease control using this regimen.

Despite controversy over the appropriate amount of radiation that may be delivered to particular areas of the skull base, the case presented here illustrates that patients may be able to tolerate high-dose radiation therapy to this area without ill effect. It is important to realize that this particular tumor was highly cellular, which may have been the reason it responded well to radiation therapy.

Steroid therapy and, when feasible, surgical debulking combined with or without low-dose radiation therapy will likely remain the standard of care in most centers. However, we believe that it may be safe and effective to offer high-dose fractionated radiotherapy in cases where steroids and surgery have been ineffective in controlling the disease process.

## Conclusions

High-dose fractionated radiotherapy is a viable alternative to steroid therapy and low-dose fractionated radiotherapy in managing inflammatory myofibroblastic tumors of the skull base in select cases.
